# EHR phenotyping via jointly embedding medical concepts and words into a unified vector space

**DOI:** 10.1186/s12911-018-0672-0

**Published:** 2018-12-12

**Authors:** Tian Bai, Ashis Kumar Chanda, Brian L. Egleston, Slobodan Vucetic

**Affiliations:** 1 Department of Computer & Information Sciences, Temple University, Philadelphia, PA USA; 2 Fox Chase Cancer Center, Temple University, Philadelphia, PA USA

**Keywords:** Electronic health records, Distributed representation, Natural language processing, Healthcare

## Abstract

**Background:**

There has been an increasing interest in learning low-dimensional vector representations of medical concepts from Electronic Health Records (EHRs). Vector representations of medical concepts facilitate exploratory analysis and predictive modeling of EHR data to gain insights about the patterns of care and health outcomes. EHRs contain structured data such as diagnostic codes and laboratory tests, as well as unstructured free text data in form of clinical notes, which provide more detail about condition and treatment of patients.

**Methods:**

In this work, we propose a method that jointly learns vector representations of medical concepts and words. This is achieved by a novel learning scheme based on the word2vec model. Our model learns those relationships by integrating clinical notes and sets of accompanying medical codes and by defining joint contexts for each observed word and medical code.

**Results:**

In our experiments, we learned joint representations using MIMIC-III data. Using the learned representations of words and medical codes, we evaluated phenotypes for 6 diseases discovered by our and baseline method. The experimental results show that for each of the 6 diseases our method finds highly relevant words. We also show that our representations can be very useful when predicting the reason for the next visit.

**Conclusions:**

The jointly learned representations of medical concepts and words capture not only similarity between codes or words themselves, but also similarity between codes and words. They can be used to extract phenotypes of different diseases. The representations learned by the joint model are also useful for construction of patient features.

## Background

Electronic health record (EHR) systems are used by medical providers to streamline the workflow and enable sharing of patient data among providers. Beyond that primary purpose, EHR data have been used in healthcare research for exploratory and predictive analytics in problems such as risk prediction [1–3] and retrospective epidemiologic studies [4–6]. Important challenges in those studies include cohort identification [7, 8], which refers to finding a set of patients receiving a specific treatment or having a specific diagnosis, and patient phenotyping [9, 10], which refers to identifying conditions and treatments for given diseases from patients’ longitudinal records.

EHR data are heterogeneous collections of both structured and unstructured information. In order to store data in a structured way, several ontologies have been developed to describe diagnoses and treatments, among which the most popular coding classification systems is the International Classification of Diseases (e.g, ICD-9, ICD-10). ICD codes provide alpha-numeric encoding of patient conditions and treatments. On the other hand, the unstructured clinical notes contain various more nuanced information (e.g, the history of patient’s illness and medication), which creates challenges for designing effective algorithms to transform data into meaningful representations that can be efficiently interpreted and used in health care applications. Various studies manage to discover knowledge from free-text clinical notes. Wang et al. proposed a token matching algorithm to map medical expressions in clinical notes into a structured medical terminology [11]. Pivovarov et al. developed a probabilistic graphical model to infer phenotypes described by medical codes, words and other clinical observations [12]. Joshi et al. proposed a non-negative matrix factorization method to generate latent factors defined by clinical words [13].

The success of extracting knowledge from clinical notes often requires application of Natural Language Processing (NLP) techniques. Learning distributed representations of words using models based on neural networks has been shown to be very useful in many NLP tasks. These models represent words as vectors and place vectors of words that occur in similar contexts in a neighborhood of each other. Among the existing models, Mikolov’s word2vec model [14] is among the most popular due to its simplicity and effectiveness in learning word representations from a large amount of data. Several studies applied word2vec on clinical notes data to produce effective clinical word representations for various applications [15–21].

While word2vec was initially designed for handling text, recent studies demonstrate that word2vec could learn representations of other types of data, including medical codes from EHR data [21–25]. Choi et al. used word2vec to learn the vector representations of medical codes using longitudinal medical records and show that the related codes indeed have similar vector representations [22]. Choi et al. designed a multi-layer perceptron to learn representations of medical codes for predicting future clinical events and clinical risk groups [23]. Gligorijevic et al. used word2vec to phenotype sepsis patients [25] and Choi et al. fed code representation learned by word2vec into a recurrent neural network to predict heart failure [24]. The limitation of those studies is that they focused only on representation of medical codes and did not utilize other sources of information from EHR data. Henriksson et al. applied word2vec to learn the vector representations of medical codes and words in clinical notes separately, and used both of them to predict adverse drug events [26, 27]. As they embed medical codes and words into two different spaces, their learned representations are not able to capture relationship between words and codes, which is exploited in our proposed method.

In this paper, we propose **JointSkip-gram** model: a novel joint learning scheme for word2vec model which embeds both diagnosis medical codes and words from clinical notes in the same continuous vector space. The resulting representations capture not only similarity between codes or words themselves, but also similarity between codes and words. We believe many clinical tasks can be viewed as measuring similarity between codes and words. For example, text-based phenotyping [12, 13] is the process of discovering the most representative words for diagnostic medical concepts. On the other hand, given a collection of words, such as clinical notes, the automatic code assignment task [11] aims to automatically assign diagnosis and procedure medical codes and thus reduce human coding effort. In this paper we illustrate that it is possible to obtain representation of words and codes in the same vector space and that the resulting representations are very informative. To achieve this objective, directly applying word2vec and related algorithms may not be appropriate since codes and words are located in different parts of EHR and have different forms and properties. Our proposed model is designed to tackle the heterogeneous nature of EHR data and build a connection between medical codes and words in clinical notes.

In our experiments, we examined if our representations are able to discover meaningful text-based phenotypes for different medical concepts. We compared our proposed model with Labeled LDA [28], a supervised counterpart of Latent Dirichlet Allocation (LDA) [29], which has been applied previously to clinical data analysis [30–32]. The results show that our representations indeed capture the relationship between words and codes. In comparison to our previous study [21], we also show that our method is able to identify common medicines and treatments for different diseases. We also construct patient representations and test the predictive power of the representations on the task of predicting patient diagnosis of the next visit given information from the current visit. The results show that representations learned by our approach outperform several baseline methods.

## Methods

After formulating the problem setup we overview Skip-gram [14], the architecture contained in word2vec toolkit designed for learning representations of natural language words, which is also the basis of our method. Then we explain the proposed JointSkip-gram model.

### Basic problem setup

Let us assume we are given a collection of patient visits. Each visit *S* is a pair (*D*,*N*), where *D* is an unordered set of medical diagnosis codes {*c*_1_,*c*_2_,*c*_3_...,*c*_*n*_} summarizing health condition of a patient and *N* is an ordered sequence of words from clinical notes recorded during the visit (*w*_1_,*w*_2_,*w*_3_...,*w*_*m*_). We denote the size of the code vocabulary *C* as |*C*| and the size of the word vocabulary *W* as |*W*|.

### Preliminary: Skip-gram

**Fig. 1:**
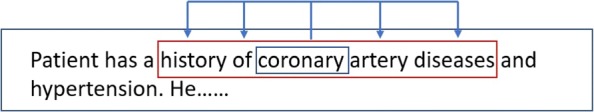
The framework of Skip-gram. Each word is used to predict its neighbours in a small context window. In this example the size of context window is 2

Figure 1 summarizes the Skip-gram framework. Given a sequence of words (*w*_1_,*w*_2_,*w*_3_...,*w*_*m*_), Skip-gram sequentially scans it. For every scanned word *w*_*i*_, called the target word, the log-likelihood of the words within its neighborhood (e.g., a window of a predefined size *q*) is calculated as

1 $$ \sum_{i-q \leq{j} \leq{i+q}, j\neq{i}} \log p(w_{j}|w_{i})  $$

where *p*(*w*_*j*_|*w*_*i*_) is the conditional probability of seeing word *w*_*j*_ as context of target word *w*_*i*_. It is defined as a softmax function

2 $$ p(w_{j}|w_{i})=\frac{e^{V_{w_{i}} \cdot U_{w_{j}}}}{\sum_{w_{k} \in W}e^{V_{w_{i}}\cdot U_{w_{k}}}}  $$

where $V_{w_{i}}$ is a *T*-dimensional vector providing the input representation of target word *w*_*i*_ and $U_{w_{j}}$ is a *T*-dimensional vector providing the context representation of context word *w*_*j*_. Skip-gram results in two matrices: the input word matrix $V\in \mathbb {R}^{|W|\times T}$ and the context word matrix $U\in \mathbb {R}^{|W|\times T}$. The obtained input word representation $V_{w_{i}}$ is typically used as word representation in downstream predictive or descriptive tasks.

To learn vector representation of words from the vocabulary, a stochastic gradient algorithm is used to maximize the objective function (1).

Maximizing (1) is computationally expensive since the denominator $\sum _{w_{k} \in W} e^{{V_{w_{i}}}{U_{w_{k}}}}$ in (2) sums over all words *w*_*k*_∈*W*. As a computationally efficient alternative of (1), Mikolov et al. proposed the skip-gram with negative sampling (SGNS) [14], which replaces log*p*(*w*_*j*_|*w*_*i*_) in (1) with the sum of two logarithmic probabilities as follows. For scanned word *w*_*i*_, the objective function becomes

3 $$ \sum_{\substack{i-q \leq{j} \leq{i+q} \\ j\neq{i}}} \left(\log p(w_{i},w_{j})+\sum_{w_{N} \in W_{neg}} \log\left(1-p(w_{i},w_{N})\right)\right)  $$

where probability *p*(*w*_*i*_,*w*_*j*_) is defined as sigmoid function $\sigma \left (V_{w_{i}}\cdot U_{w_{j}}\right)$:

4 $$ p(w_{i},w_{j})=\sigma\left(V_{w_{i}}\cdot U_{w_{j}}\right)=\frac{1}{1+e^{-V_{w_{i}}\cdot U_{w_{j}}}}  $$

and *W*_*neg*_={*w*^*k*^∼*P*_*w*_|*k*=1,...,*K*} is the set of so-called “negative words” that are sampled from the marginal distribution *P*_*w*_ of words. *K* is a hyperparameter determining the number of negative words generated with each context word. The assumption is that words sampled from the marginal distribution are less likely to co-occur as context of the target word. The first term of (3) is the probability that two words occur as target and context in the data set, while the second term of (3) is the probability that a target word and “negative words” in *W*_*neg*_ are not observed co-occurring in the dataset. By maximizing (3), the dot product between frequently co-occurring words would become large while the dot product between rarely co-occurring words would become small. In other words, in the resulting *T*-dimensional vector space, the related words will be placed in the vicinity of each other, such that their cosine similarity is high.

### Proposed model: JointSkip-gram

In the Skip-gram model, each scanned word is used to predict probability of its neighboring words in the sequence. However, in the electronic health records each visit consists of clinical notes, which are ordered sequences of words, and medical codes, which are sets. We are interested in jointly learning vector representation of words and codes in the same vector space. Both medical codes and clinical notes describe condition and treatment of a patient and they are closely related. For example, if a patient is assigned ICD-9 code “174” (female breast neoplasm), the corresponding clinical notes are likely to mention surgery (e.g, mastectomy or lumpectomy). To derive JointSkip-gram, we first need to define context of each word and each code.

**Fig. 2:**
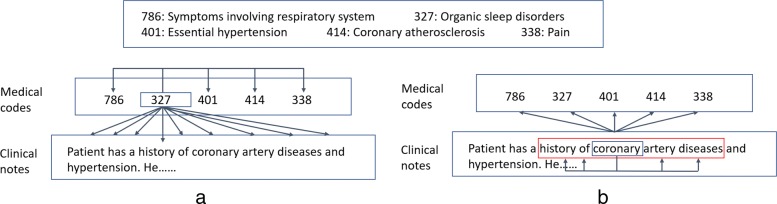
The framework of JointSkip-gram. **a** Each code is used to predict all other codes and words in the same visit. **b** Each word is used to predict all codes in the same visit and its neighbour words in a small context window to keep its syntactic properties

Since the codes are unordered, we define the context of target code *c*_*i*_ as all other codes in the same visit, as well as all words in the clinical note. Thus, as shown in Fig. 2a, in JointSkip-gram, every scanned code *c*_*i*_ is used to predict other codes in *D* and all words in *N*. The log-likelihood of code *c*_*i*_ can be expressed as

5 $$ \sum_{\substack{1 \leq{j} \leq{n} \\ j\neq{i}}} \log p(c_{j}|c_{i})+\sum_{1 \leq{j} \leq{m}} \log p(w_{j}|c_{i})  $$

Similarly to Skip-gram, the probabilities *p*(*c*_*j*_|*c*_*i*_) and *p*(*w*_*j*_|*c*_*i*_) are defined as softmax functions

6 $$ p(c_{j}|c_{i})=\frac{e^{V_{c_{i}}\cdot U_{c_{j}}}}{\sum_{c_{k} \in C}e^{V_{c_{i}}\cdot U_{c_{k}}}}  $$

and

7 $$ p(w_{j}|c_{i})=\frac{e^{V_{c_{i}}\cdot U_{w_{j}}}}{\sum_{w_{k} \in W}e^{V_{c_{i}}\cdot U_{w_{k}}}}  $$

For words in clinical notes we define two types of contexts. One consists of neighboring words in the note. Another consists of all codes in the medical code set. Thus, as shown in Fig. 2b, for scanned word *w*_*i*_ in *N* JointSkip-gram uses words within a window of a predefined size *q* as its context words. It also uses all codes in *D* as its context codes. The resulting log-likelihood of word *w*_*i*_ can be expressed as

8 $$  \sum_{\substack{i-q \leq{j} \leq{i+q} \\ j\neq{i}}} \log p(w_{j}|w_{i})+\sum_{1 \leq{j} \leq{n}} \log p(c_{j}|w_{i})  $$

in which

9 $$ p(w_{j}|w_{i})=\frac{e^{V_{w_{i}}\cdot U_{w_{j}}}}{\sum_{w_{k} \in W}e^{V_{w_{i}}\cdot U_{w_{k}}}}  $$

and

10 $$ p(c_{j}|w_{i})=\frac{e^{V_{w_{i}}\cdot U_{c_{j}}}}{\sum_{c_{k} \in C}e^{V_{w_{i}}\cdot U_{c_{k}}}}  $$

Maximizing the sum of objective functions (5) and (8) over the whole data set of visits is computationally expensive since in (6), (7), (9) and (10), the denominators sum over all words in *W* and all codes in *C*. Similar to SGSN [14], we use a computationally cheaper algorithm that relies on negative sampling. Instead of calculating the softmax function, the negative sampling approach uses computationally inexpensive sigmoid function to represent the probability that a word or a code is within a context of a target word or a code. For each scanned code *c*_*i*_, the negative sampling objective function becomes

11 $${}\begin{aligned} &\sum_{\substack{1 \leq{j} \leq{n} \\ j\neq{i}}} \left(\log p\left(c_{i},c_{j}\right)+\sum_{c_{N} \in C_{neg}} \log\left(1-p\left(c_{i},c_{N}\right)\right)\right) \\ &+\sum_{1 \leq{j} \leq{m}} \left(\log p\left(c_{i},w_{j}\right)+\sum_{w_{N} \in W_{neg}} \log\left(1-p\left(c_{i},w_{N}\right)\right)\right) \end{aligned}  $$

where

12 $$ p\left(c_{i},c_{j}\right)=\sigma\left(V_{c_{i}}\cdot U_{c_{j}}\right)=\frac{1}{1+e^{-V_{c_{i}}\cdot U_{c_{j}}}}  $$

and

13 $$ p\left(c_{i},w_{j}\right)=\sigma\left(V_{c_{i}}\cdot U_{w_{j}}\right)=\frac{1}{1+e^{-V_{c_{i}}\cdot U_{w_{j}}}}  $$

*C*_*neg*_={*c*^*k*^∼*P*_*c*_|*k*=1,...,*K*} is the set of “negative codes” that are sampled from marginal distribution *P*_*c*_ of codes and *W*_*neg*_={*w*^*k*^∼*P*_*w*_|*k*=1,...,*K*} is the set of negative words that are sampled from a marginal distribution *P*_*w*_ of words, where *K* is the number of negative samples.

Similarly, for each scanned word *w*_*i*_, the negative sampling objective criterion becomes:

14 $${}\begin{aligned} &\sum_{\substack{i-q \leq{j} \leq{i+q} \\ j\neq{i}}} \left(\log p\left(w_{i},w_{j}\right)+\sum_{w_{N} \in W_{neg}} \log\left(1-p\left(w_{i},w_{N}\right)\right)\right) \\ &+\sum_{1 \leq{j} \leq{n}} \left(\log p\left(w_{i},c_{j}\right)+\sum_{c_{N} \in C_{neg}} \log\left(1-p\left(w_{i},c_{N}\right)\right)\right) \end{aligned}  $$

where

15 $$ p\left(w_{i},w_{j}\right)=\sigma\left(V_{w_{i}}\cdot U_{w_{j}}\right)=\frac{1}{1+e^{-V_{w_{i}}\cdot U_{w_{j}}}}  $$

and

16 $$ p\left(w_{i},c_{j}\right)=\sigma\left(V_{w_{i}}\cdot U_{c_{j}}\right)=\frac{1}{1+e^{-V_{w_{i}}\cdot U_{c_{j}}}}  $$

*C*_*neg*_ and *W*_*neg*_ are the same as in (11). By maximizing (14), the probabilities *p*(*w*_*i*_,*w*_*j*_) and *p*(*w*_*i*_,*c*_*j*_) of related words and codes will be large.

Similarly to Skip-gram, stochastic gradient descent algorithm is applied in jointSkip-gram to learn vector representations of codes and words that maximize (11) and (14). The input vector representation matrix *V* is used as the resulting representation of words and codes. Since we jointly learn vector representations of codes and words, matrices $V\in \mathbb {R}^{\left (|W|+|C|\right)\times T}$ and $U\in \mathbb {R}^{\left (|W|+|C|\right)\times T}$ include representations of both words and codes. In the resulting vector space, similarity of two vectors is measured using cosine similarity. The vectors of similar codes or words should be close to each other. Since JointSkip-gram represents codes and words in the same vector space, the words related to a given medical code should be placed in vicinity.

## Results

### Dataset description

**MIMIC-III Dataset**: The MIMIC-III Critical Care Database [33] is a publicly-available database which contains de-identified health records of 46,518 patients who stayed in the Beth Israel Deaconess Medical Center’s Intensive Units from 2001 to 2012. Each visit in the dataset contains both structured health records data and free text clinical notes.

We used EHR data from all patients in the dataset. The total number of patient visits in MIMIC-III is 58,597. On average, each patient had 1.26 visits, 38,991 patients had a single visit, 5151 had two visits, and 2376 patients had 3 or more visits. The average number of the recorded ICD-9 diagnosis codes per visit is 11 and the average number of words in clinical notes is 7898. For each patient visit, we extracted all diagnosis codes and all clinical notes.

**Preprocessing**: For each EHR in the dataset we are only focusing on the clinical notes and ICD-9 diagnosis codes. Each clinical note was preprocessed in the following way. All digits and stop words were removed. The typos were filtered using a standard English vocabulary in PyEnchant, a Python library for spell checking. For representation learning, rare words were filtered out since they do not appear often enough to obtain good quality representations. Therefore, all words whose frequency is less than 50 were removed. The resulting number of unique words was 14,302. Furthermore, the total number of unique ICD-9 diagnosis codes in MIMIC-III is 6984. Codes whose frequency is less than 5 were removed. This reduced the number of codes to 3874. Since some codes were still relatively rare for learning meaningful representations, we exploited the hierarchical tree structure of ICD-9 codes and grouped them by their first three digits. For example, ICD-9 codes “2901” (presenile dementia), “2902” (senile dementia with delusional or depressive) and “2903” (senile dementia with delirium) were grouped into a single code “290” (dementias). The size of the final code vocabulary was 752.

**Training and Test Patients**: We randomly split the patients into training and test sets. All 38,991 patients with a single visit were placed in the training set. Of the 7527 patients with 2 or more visits, we randomly assigned 80% of them (6015 patients) to the training set and 20% of them (1512 patients) to the test set. The whole training set was used for learning of vector representations. We excluded patients with only a single visit for the task of next visit prediction because this task requires patients to have at least two visits.

### Training JointSkip-gram model

EHRs of patients from the training set were used to learn our JointSkip-gram model. For each visit we created a (*D*,*N*) pair. There were 54,965 such pairs in the training data. The size *T* of vectors representing codes and words was set to 200. Stochastic gradient algorithm with negative sampling maximizing (11) and (14) was set to loop through all the training data 40 times because we empirically observed that it was sufficient for the algorithm to converge. The number of negative samples was set to 5 and the size of the window for word context in the clinical notes was set to 5. As a result, each of the 7898 words and 752 ICD-9 codes were represented as 200-dimensional vectors in a joint vector space. Before applying JointSkip-gram model, we used a small fraction (∼10%) of clinical notes to pretrain vector representations of words only, as we observed that this improves our final representations.

To evaluate the quality of vector representations, we performed two types of experiments: (1) phenotype and treatment discovery by evaluating associations between codes and words in the vector space, (2) testing the predictive power of the vector representations on the task of predicting medical codes of the next visit.

### Phenotype discovery

Text-based phenotype discovery can be viewed as finding words representative of medical codes. For a given ICD-9 diagnosis code, we retrieved its nearest 15 words in the vector space. If successful, the neighboring words should be clinically relevant to the ICD-9 code.

As an alternative to JointSkip-gram, we used **labeled latent Dirichlet allocation** (LLDA) [28], a supervised version of LDA [29]. In LLDA, there is a one-to-one correspondence between topics and labels. LLDA assumes there are multiple labels associated with each document and assigns each word a probability that it corresponds to each label. LLDA can be naturally adapted to our case by treating medical codes as labels and clinical notes as documents. For a given ICD-9 diagnosis code we retrieved 15 words with the highest probabilities and compared those words with the 15 words obtained by JointSkip-gram.

We consulted domain experts about quality of the extracted phenotypes. First, we selected 6 diverse ICD-9 codes from MIMIC-III that cover both acute and chronic diseases and both common and less common conditions. The 6 ICD-9 codes are listed in Table 1, together with their description and frequency in the training set. Table 1 shows the list of 15 closest words by both methods to the 6 ICD-9 codes. For each ICD-9 diagnosis code, we presented the two lists in a random order to a medical expert and asked two questions: (1) which list is a better representative of the diagnosis code, and (2) which words in each list are not highly related to the given diagnosis code. We recruited four physicians from the Fox Chase Cancer Center as medical experts for the evaluation.

**Table 1:** Most important 15 words (ranked by importance) for ICD-9 codes “570”, “174”, “295”, “348”, “311”, “042”

**570 (Acute liver failure, 1067)**		**174 (Female breast cancer, 139)**	
**JointSkip-gram**	**LLDA**	**JointSkip-gram**	**LLDA**
Liver	Arrest	Metastatic	Breast
Hepatic	Pea	Mets	Pres
Cirrhosis	Cooling	Cancer	Mastectomy
Rising	Sun	Breast	Flap
Markedly	Arctic	Metastases	Mets
Shock	Rewarmed	Malignant	Ca
Lactate	Cooled	Metastasis	Cancer
Encephalopathy	Atrophine	Oncologist	Metastatic
Amps	Dopamine	Oncology	Chemotherapy
Picture	Rewarming	Chemotherapy	Malignant
Rise	Cardiac	Infiltrating	Oncologist
Elevated	Coded	Palliative	Polumoprhic
Cirrhotic	Continue	Tumor	Reversible
Bicarb	Prognosis	Melanoma	Mastectomies
AQlcoholic	Ems	Mastectomy	Crisis
**295 (Schizophrenic disorders, 691)**		**348 (Conditons of brain, 3781)**	
**JointSkip-gram**	**LLDA**	**JointSkip-gram**	**LLDA**
Schizophrenia	Schizophrenia	Hemorrhagic	Arrest
Psych	Paranoid	Herniation	Herniation
Bipolar	Psych	Temporal	Unresponsive
Suicide	Psychiatric	Cerebral	Corneal
Psychiatry	Disorders	Brain	Pupils
Kill	Personality	Hemorrhage	Brain
Paranoid	Hiss	Parietal	Cooling
Ideation	Guardian	Ganglia	Posturing
Psychiatrist	Psychiatry	Occipital	Head
Hallucinations	Hypothyroidism	Extension	Nemorrhage
Psychosis	Home	Surrounding	Noxious
Personality	Aloe	Head	Family
Sitter	Arrest	Effacement	Prognosis
Disorder	Pt	Ataxia	Pea
Abuse	Unresponsive	Burr	Gag
**311 (Depressive disorder, 3431)**		**042 (HIV, 538)**	
**JointSkip-gram**	**LLDA**	**JointSkip-gram**	**LLDA**
Patient	Depression	Aids	Aids
Abuse	Tablet	Viral	Immunodeficiency
Hallucinations	Blood	Fungal	Virus
Withdrawal	Daily	Opportunistic	Human
Ingestion	Campus	Bacterial	Viral
Questionable	Mg	Disseminated	Load
Thiamine	Garage	Immuno-deficiency	Cooling
Remote	Capsule	Tuberculosis	Partner
Alcohol	Building	Organisms	Acyclovir
Significant	Parking	Herpes	Thrush
Overdose	One	Undetectable	Fevers
Prior	Discharge	Acyclovir	Induced
Apparent	Normal	Detectable	Antigen
Depression	East	Chlamydia	Pneumonia
Although	Coherent	Syphilis	Blanket

Disease description and frequency are listed in the brackets

The evaluation results are summarized in Table 2. As could be seen, all 4 experts agreed that JointSkip-gram words better represent ICD-9 codes 570, 348, and 311. For the remaining 3 codes (174, 295, 042), the experts were split, but in no case the majority preferred the LLDA words. By considering the average number of words deemed unrelated by the experts, the experts found that JointSkip-gram was superior to LLDA for all 6 ICD-9 diagnosis codes.

**Table 2:** Evaluation results by clinical experts

**# of experts who think the method is better than the other**						
**ICD-9 codes**	**570**	**174**	**295**	**348**	**311**	**042**
JointSkip-gram	4	2	3	4	4	2
LLDA	0	2	1	0	0	2
**Average # of unrelated words across experts**						
**ICD-9 codes**	**570**	**174**	**295**	**348**	**311**	**042**
JointSkip-gram	2.25	0.75	0.75	1.25	3.25	0.75
LLDA	9.25	1.75	3	3.75	6.5	2.75

For ICD-9 code “570” (acute liver failure), JointSkip-gram finds “liver”, “hepatic”, “cirrhosis”, which are directly related to acute liver failure. Remaining words in the JointSkip-gram list are mostly indirectly related to liver failure, such as “alcoholic”, which explains one of the primary reasons for liver damage. On the other hand, LLDA captured a few related words, as evidenced by an average of 9.25 words that experts found unrelated. Among those unrelated words we find “cooling”, “sun”, “arctic”, “rewarmed”, “cooled”, “rewarming”, “coded”, “continue”, and “prognosis”.

For ICD-9 code “174” (female breast cancer), “295” (Schizopherenic disorders) and “042” (HIV), both Joint-Skipgram and LLDA find highly related words. One of our experts commented that several words found by JointSkip-gram are diseases which are likely to co-occur with the given disease. For example, JointSkip-gram finds “melanoma” for female breast cancer and “herpes”, “chlamydia”, “syphilis” for HIV. This suggests that JoinSkip-gram captures the hidden relationships between diseases, which could make it suitable for understanding of comorbidities.

For code “311” (depressive disorder), both JointSkip-gram and LLDA had difficulties in finding related words. According to feedback from one of our experts, “abuse”, “hallucinations”, “alcohol”, “overdose”, “depression” and “thiamine” (note: depression is a common symptom of thiamine deficiency) found by JointSkip-gram are related to the disease, while only “depression”, “tablet”, “capsule” found by LLDA are recognizably related to depression. We hypothesize that for common diseases (e.g, “depression” and “hypertension”), which are rarely the primary diagnosis or a major factor in deciding an appropriate treatment of the main condition, physicians rarely discuss them in clinical notes. Thus, it is difficult for any algorithm to discover words from clinical notes related to such diagnoses.

### Treatment discovery

In our preliminary study [21], we used PyEnchant standard English vocabulary to filter out the typos in clinical notes. However, there are many nonstandard English terms used in medical notes to describe medical treatments, medicines, and diagnoses. These nonstandard words are not part of PyEnchant standard English vocabulary we used for preprocessing, but they could have important meaning. Hence, we repeated our experiments by including all words occurring more than 50 times. The resulting vocabulary increased to 33,336 unique words.

After running our Joint-Skipgram model on the new dataset, we looked at the representative words for each diagnosis code. Tables 3 and 4 show the 15 nearest clinical note words in the vector space to ICD-9 codes “570” and “174”, respectively. We can observe that many retrieved words are different from those in Table 1 for codes “570” and “174”. The words that also appear in Table 1 are marked with italic font in Tables 3 and 4.

**Table 3:** Most important 15 words (including nonstandard English words) (ranked by importance) for ICD-9 codes “570”

**ICD-9: 570 (Acute liver failure)**	
**Word**	**Description**
*liver*	An organ that produces biochemicals necessary for digestion
Renal	Relating to the kidneys
Hepatorenal	A life-threatening medical condition that consists of rapid deterioration in kidney
Crrt	CRRT is a dialysis modality used to treat critically ill, hospitalized patients
Vasopressin	A hormone synthesized
*Shock*	Shock liver is a condition defined as an acute liver injury
Failure	Liver failure can occur gradually
Levophed	Injection
Ascites	Ascites is the abnormal buildup of fluid in the abdomen
Oliguric	A urine output
Pigtail	Pigtail drainage is used for liver abscess
Transplant	liver transplant is a surgical procedure
Rifaximin	Antibiotic
*Cirrhosis*	Cirrhosis is a late stage of scarring (fibrosis) of the liver
*Hepatic*	Relating to the liver.

**Table 4:** Most important 15 words (including nonstandard English words) (ranked by importance) for ICD-9 codes 174

**ICD-9: 174 (Female breast cancer)**	
**Word**	**Description**
Xeloda	A prescription medicine used to treat people with cancer
Tamoxifen	A medication that is used to prevent breast cancer
*Metastatic*	A pathogenic agent’s spread from an primary site to a different site
*Chemotherapy*	A treatment by the use of chemical substances
*Cancer*	A disease in which abnormal cells divide uncontrollably and destroy body tissue
Carboplatin	It is used to treat ovarian cancer
Onc	Abbreviations of oncologist
*Oncologist*	A doctor who treats cancer
Taxol	It belongs to a class of chemotherapy drugs is the abnormal buildup of fluid in the abdomen
Chemo	Short form of chemotherapy
Gemcitabine	Gemcitabine is an anti-cancer
*Mets*	Abbreviations of metastasis
Compazine	This medication is used to treat severe nausea
*Palliative*	A medical care for relieving pain
Metastases	The development of secondary malignant growths

A close look into Tables 3 and 4 reveals that most neighbors are specific medical terminology words describing drugs or treatments related to the diagnosis. For example, words “crrt”, “levophed”, “rifaximin”, and “transplant” in Table 3, are related to treatment of acute liver failure. Similarly, words “xcloda”, “tamoxifen”, “carboplatin”, “taxol”, “compazine” in Table 4 are related to cancer treatment. Therefore, including nonstandard words in our vocabulary enabled us to connect specialized medical terms with particular ICD-9 diagnosis codes.

### Predictive evaluation

In another group of experiments we constructed patient representations and evaluated quality of the vector representations of words and medical codes through predictive modeling. We adopted the evaluation approach used in [34], which predicts medical codes of the next visit given the information from the current visit. Specifically, given two consecutive visits of a patient, we used information of the first visit (i.e., medical codes and clinical notes) to predict medical codes assigned during the second visit. In the previous work on this topic, the authors of [23, 34, 35] used medical codes as features for prediction. In our evaluation, we used both medical codes and clinical notes to create predictive features. To generate a feature vector for the first visit, we found the average JointSkip-gram vector representation of the diagnosis codes and the average JointSkip-gram vector representation of the words used in clinical notes. Then, we concatenated those two averaged vectors. We call this method **Concatenation-JointSG** and compare it with the following five baselines:

**Concatenation-One**: The one-hot vector of medical codes and the one-hot vector of clinical notes for a given visit were concatenated. In the one-hot vector of each visit, words and codes which occur in the visit were encoded as 1, otherwise they were encoded as 0.

**SVD**: Singular vector decomposition (SVD) was applied to Concatenation-One representations to generate dense representations of visits.

**LDA**: Using latent Dirichlet allocation (LDA) [29], each document was represented as a topic probability vector. This vector was used as the visit representation. To apply LDA, for each visit we created a document that consists of concatenation of a list of medical diagnosis codes and clinical notes. We note that LLDA is not suitable for this task since its topics only contain words.

**Codes-JointSG**: To evaluate the predictive power of medical codes, we created features for a visit as the average JointSkip-gram vector representation of the diagnosis codes.

**Words-JoinSG**: To evaluate the predictive power of clinical notes, we created features for a visit as the average JointSkip-gram vector representation of the words in clinical notes.

To compare vector representations obtained by JointSkip-gram and Skip-gram, we also trained Skip-gram on clinical notes and on medical codes separately. The resulting vector representations are not in the same vector space. We used Skip-gram representations to construct 3 more groups of features:

**Codes-SG**: The features for a visit were the average Skip-gram vector representation of the diagnosis codes.

**Words-SG**: The features for a visit were the average Skip-gram vector representation of the words in clinical notes.

**Concatenation-SG**: We concatenated the features from Codes-SG and Words-SG.

Given a set of features describing the first visit, we used softmax to predict medical codes of the second visit. Let us assume the feature vector of the first visit is *x*_*t*_, the size of code vocabulary is |*C*| and $Z\in \mathbb {R}^{(|C| \times |x_{t}|)}$ is the weight matrix of softmax function. The probability that the next visit *y*_*t*+1_ contains medical code *c*_*i*_ is calculated as

$$p(y_{t+1}(c_{i})=1)=\frac{e^{Z_{i} \cdot x_{t}}}{\sum_{c_{k} \in C}e^{Z_{k} \cdot x_{t}}} $$

We use Top-k recall [34] to measure the predictive performance, because it mimics the behavior of doctors who list the most probable diagnoses upon observation of a patient. For each visit, softmax recommends *k* codes with the highest probabilities and Top-k recall is calculated as

$$\text{Top-k recall}=\frac{\text{the number of true positives in } {k} \text{ codes}}{\text{the number of all positives}} $$

In the experiment, we tested Top-k recall when *k*=20, *k*=30, and *k*=40.

**Training details**: To create features for all proposed models (Skip-gram, JointSkip-gram, LDA, SVD), we used the training set. To train the Skip-gram model, we used 40 iterations, 5 negative samples, and the window size 5 (the same as for JointSkip-gram). For SVD and LDA, we set the maximum number of iterations to 1000 to guarantee convergence. For JointSkip-gram, Skip-gram, SVD and LDA, we set the dimensionality of feature vectors to 200.

To train the softmax model, we created the labeled set using only patients with 2 or more visits. We sort all visits of each such patient by the admission time. Given two consecutive visits, we use the former to create features and the latter to create the labels. As a result, the labeled set used to train the softmax model had 9955 labeled examples and the test set had 2489 labeled examples. The softmax model for prediction was trained for 100 epochs using a stochastic gradient algorithm to minimize the categorical cross entropy loss.

Table 5 shows the performance of softmax models that use different sets of features. A model using Concatenation-JointSG features outperformed other baselines on all three Top-k measures.

**Table 5:** Performance of predicting medical codes of the next visit

Model	Top-20 recall	Top-30 recall	Top-40 recall
Concatenation-One	0.489 ±0.004	0.590 ±0.004	0.661 ±0.004
SVD	0.478 ±0.004	0.588 ±0.004	0.652 ±0.004
LDA	0.431 ±0.004	0.530 ±0.004	0.605 ±0.004
Codes-JointSG	0.499 ±0.003	0.592 ±0.003	0.662 ±0.003
Words-JointSG	0.437 ±0.004	0.536 ±0.004	0.609 ±0.004
Concatenation-JointSG	**0.506 ±0.003**	**0.599 ±0.003**	**0.670 ±0.003**

The average and standard error of Top-k recall (k=20, 30, 40) are provided

## Discussion

### Predictive evaluation analysis

The results in Table 5 not only show the advantage of our model, but also demonstrate that both medical codes and clinical notes in Concatenation-JointSG contributed to the prediction of future visit, since using the concatenation of word representations and code representations outperformed both Codes-JointSG and Words-JointSG. While Codes-JointSG achieved considerably high recall, Words-JointSG performed relatively worse. The lower accuracy of Words-JointSG likely indicates that using the average of word vectors might not be the best strategy to use clinical note information. A future direction could be to use a neural network (NN) such as convolutional NN or recurrent NN to better capture information contained in clinical notes.

**Fig. 3:**
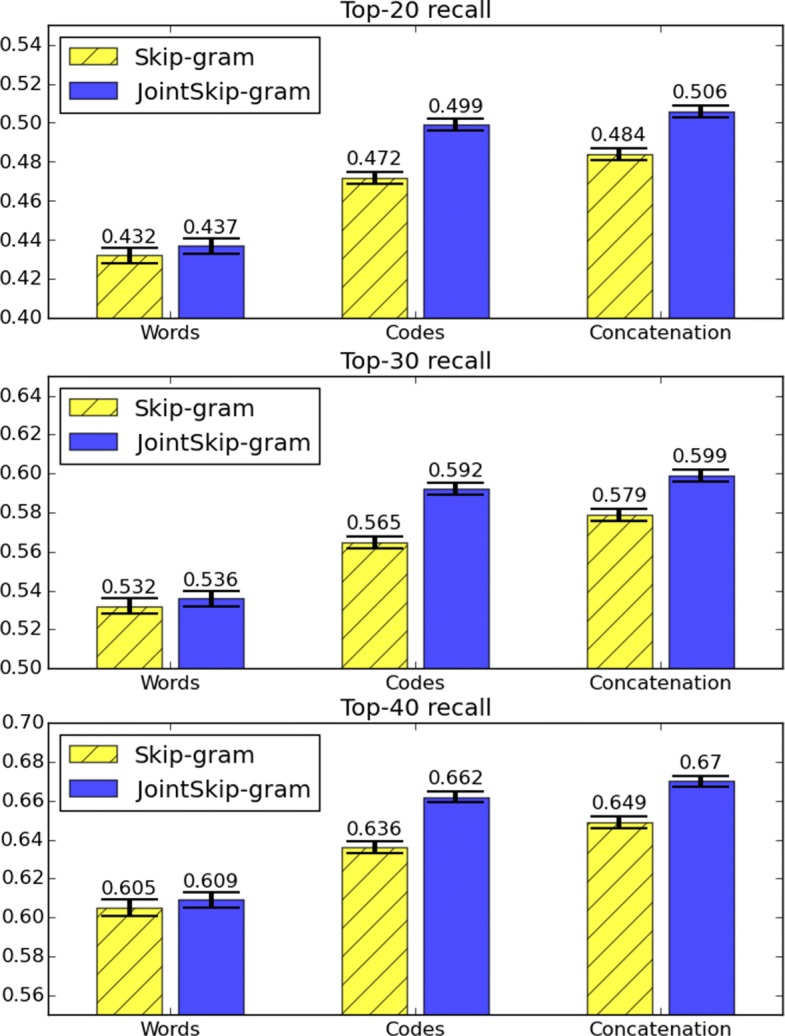
Top-k recall (k=20, 30 and 40) for JointSkip-gram and Skip-gram. The error bars indicate the standard error

Figure 3 shows comparison between JointSkip-gram and Skip-gram features. From the figure, we can observe that features generated by JointSkip-gram outperformed those generated by Skip-gram. While the difference between Words-JointSG and Words-SG were not large, Codes-JointSG and Concatenation-JointSG significantly outperformed Codes-SG and Concatenation-SG, respectively. This strongly indicates that JointSkip-gram not only captures the relationship between medical codes and words, but also learns improved word and code representations.

### Limitations and future works

One limitation of our work is that in processing step we removed words whose frequency are less than 50 and codes whose frequency are less than 5. We also grouped all codes by their first three digits because rare codes are not statistically significant enough to learn meaningful representations. One way to use rare tokens is to exploit the domain knowledge such as subword information or hierarchical tree structure of medical codes.

The future work should consider applying joint representations to a broader range of tasks, such as cohort identification and automatic code assignment. It would also be interesting to explore more advanced prediction models such as deep neural networks.

## Conclusions

In this paper, we proposed JointSkip-gram algorithm to jointly learn representation of words from clinical notes and diagnosis codes in EHR. JointSkip-gram exploits the relationship between diagnosis codes and clinical notes in the same visit and represents them in the same vector space. The experimental results demonstrate that the resulting code and word representation can be used to discover meaningful disease phenotypes. They also indicate that the representations learned by the joint model are useful for construction of patient features.

## Abbreviations

EHR: Electronic health record
ICD: International classification of diseases
LDA: Latent Dirichlet allocation
LLDA: Labeled latent Dirichlet allocation
NLP: natural language processing
NN: neural network
SVD: Singular vector decomposition

## References

[1] Yan Y, Birman-Deych E, Radford MJ, Nilasena DS, Gage BF (2005). Comorbidity indices to predict mortality from medicare data: results from the national registry of atrial fibrillation. Med Care.

[2] Krumholz HM, Wang Y, Mattera JA, Wang Y, Han LF, Ingber MJ, Roman S, Normand S-LT (2006). An administrative claims model suitable for profiling hospital performance based on 30-day mortality rates among patients with heart failure. Circulation.

[3] Klabunde CN, Potosky AL, Legler JM, Warren JL (2000). Development of a comorbidity index using physician claims data. J Clin Epidemiol.

[4] Levitan N, Dowlati A, Remick S, Tahsildar H, Sivinski L, Beyth R, Rimm A (1999). Rates of initial and recurrent thromboembolic disease among patients with malignancy versus those without malignancy. Risk Anal Medicare Claims Data. Med (Baltimore).

[5] Taylor Jr DH, Østbye T, Langa KM, Weir D, Plassman BL (2009). The accuracy of medicare claims as an epidemiological tool: the case of dementia revisited. J Alzheimers Dis.

[6] Schneeweiss S, Seeger JD, Maclure M, Wang PS, Avorn J, Glynn RJ (2001). Performance of comorbidity scores to control for confounding in epidemiologic studies using claims data. Am J Epidemiol.

[7] Nattinger AB, Laud PW, Bajorunaite R, Sparapani RA, Freeman JL (2004). An algorithm for the use of medicare claims data to identify women with incident breast cancer. Health Serv Res.

[8] Winkelmayer WC, Schneeweiss S, Mogun H, Patrick AR, Avorn J, Solomon DH (2005). Identification of individuals with ckd from medicare claims data: a validation study. Am J Kidney Dis.

[9] Warren JL, Klabunde CN, Schrag D, Bach PB, Riley GF. Overview of the seer-medicare data: content, research applications, and generalizability to the united states elderly population. Med Care. 2002;40:3–18. 10.1097/01.MLR.0000020942.47004.03 12187163

[10] Halpern Y, Horng S, Choi Y, Sontag D (2016). Electronic medical record phenotyping using the anchor and learn framework. J Am Med Inform Assoc.

[11] Wang Y, Patrick J (2008). Mapping clinical notes to medical terminology at point of care. Proceedings of the Workshop on Current Trends in Biomedical Natural Language Processing.

[12] Pivovarov R, Perotte AJ, Grave E, Angiolillo J, Wiggins CH, Elhadad N (2015). Learning probabilistic phenotypes from heterogeneous ehr data. J Biomed Inform.

[13] Joshi S, Gunasekar S, Sontag D, Ghosh J. Identifiable phenotyping using constrained non-negative matrix factorization; 2016, pp. 17–41. arXiv preprint arXiv:1608.00704.

[14] Mikolov T, Sutskever I, Chen K, Corrado GS, Dean J. Distributed representations of words and phrases and their compositionality. In: Advances in Neural Information Processing Systems.2013. p. 3111–9.

[15] Moen H, Ginter F, Marsi E, Peltonen L-M, Salakoski T, Salanterä S. Care episode retrieval: distributional semantic models for information retrieval in the clinical domain. In: BMC Medical Informatics and Decision Making, vol. 15. BioMed Central: 2015. p. 2. 10.1186/1472-6947-15-S2-S2. PMC4474584 26099735

[16] Wu Y, Xu J, Jiang M, Zhang Y, Xu H. A study of neural word embeddings for named entity recognition in clinical text. In: AMIA Annual Symposium Proceedings, vol. 2015. American Medical Informatics Association: 2015. p. 1326. PMC4765694 26958273

[17] De Vine L, Zuccon G, Koopman B, Sitbon L, Bruza P (2014). Medical semantic similarity with a neural language model. Proceedings of the 23rd ACM International Conference on Conference on Information and Knowledge Management.

[18] Amunategui M, Markwell T, Rozenfeld Y. Prediction using note text: Synthetic feature creation with word2vec; 2015. arXiv preprint arXiv:1503.05123.

[19] Ghassemi MM, Mark RG, Nemati S. A visualization of evolving clinical sentiment using vector representations of clinical notes. In: Computing in Cardiology Conference (CinC), 2015. IEEE: 2015. p. 629–32. 10.1109/CIC.2015.7410989. PMC5070922 27774487

[20] Henriksson A. Representing clinical notes for adverse drug event detection. In: Proceedings of the Sixth International Workshop on Health Text Mining and Information Analysis.2015. p. 152–8.

[21] Bai T, Chanda AK, Egleston BL, Vucetic S. Joint learning of representations of medical concepts and words from ehr data. In: Bioinformatics and Biomedicine (BIBM), 2017 IEEE International Conference On. IEEE: 2017. p. 764–9. 10.1109/BIBM.2017.8217752. PMC5783648 29375929

[22] Choi Y, Chiu CY-I, Sontag D (2016). Learning low-dimensional representations of medical concepts. AMIA Summits Transl Sci Proc.

[23] Choi E, Bahadori MT, Searles E, Coffey C, Thompson M, Bost J, Tejedor-Sojo J, Sun J (2016). Multi-layer representation learning for medical concepts. Proceedings of the 22nd ACM SIGKDD International Conference on Knowledge Discovery and Data Mining.

[24] Choi E, Schuetz A, Stewart WF, Sun J (2016). Using recurrent neural network models for early detection of heart failure onset. J Am Med Inform Assoc.

[25] Stojanovic J, Gligorijevic D, Radosavljevic V, Djuric N, Grbovic M, Obradovic Z (2017). Modeling healthcare quality via compact representations of electronic health records. IEEE/ACM Trans Comput Biol Bioinforma (TCBB).

[26] Henriksson A, Zhao J, Boström H, Dalianis H. Modeling electronic health records in ensembles of semantic spaces for adverse drug event detection. In: Bioinformatics and Biomedicine (BIBM), 2015 IEEE International Conference On. IEEE: 2015. p. 343–50. 10.1109/BIBM.2015.7359705.

[27] Henriksson A, Zhao J, Dalianis H, Boström H (2016). Ensembles of randomized trees using diverse distributed representations of clinical events. BMC Med Inform Decis Mak.

[28] Ramage D, Hall D, Nallapati R, Manning CD (2009). Labeled lda: A supervised topic model for credit attribution in multi-labeled corpora. Proceedings of the 2009 Conference on Empirical Methods in Natural Language Processing: Volume 1-Volume 1.

[29] Blei DM, Ng AY, Jordan MI (2003). Latent dirichlet allocation. J Mach Learn Res.

[30] Chan KR, Lou X, Karaletsos T, Crosbie C, Gardos S, Artz D, Ratsch G. An empirical analysis of topic modeling for mining cancer clinical notes. In: Data Mining Workshops (ICDMW), 2013 IEEE 13th International Conference On. IEEE: 2013. p. 56–63. 10.1109/ICDMW.2013.91.

[31] Arnold CW, El-Saden SM, Bui AA, Taira R. Clinical case-based retrieval using latent topic analysis. In: AMIA Annual Symposium Proceedings, vol. 2010. American Medical Informatics Association: 2010. p. 26. PMC3041464 21346934

[32] Ghassemi M, Naumann T, Doshi-Velez F, Brimmer N, Joshi R, Rumshisky A, Szolovits P (2014). Unfolding physiological state: Mortality modelling in intensive care units. Proceedings of the 20th ACM SIGKDD International Conference on Knowledge Discovery and Data Mining.

[33] Johnson AE, Pollard TJ, Shen L, Li-wei HL, Feng M, Ghassemi M, Moody B, Szolovits P, Celi LA, Mark RG (2016). Mimic-iii, a freely accessible critical care database. Sci Data.

[34] Choi E, Bahadori MT, Schuetz A, Stewart WF, Sun J. Doctor ai: Predicting clinical events via recurrent neural networks. In: Machine Learning for Healthcare Conference: 2016. p. 301–18. PMC5341604 28286600

[35] Esteban C, Staeck O, Baier S, Yang Y, Tresp V. Predicting clinical events by combining static and dynamic information using recurrent neural networks. In: Healthcare Informatics (ICHI), 2016 IEEE International Conference On. IEEE: 2016. p. 93–101.

